# Antioxidant, antimicrobial, toxicity and analgesic properties of ethanol extract of *Solena amplexicaulis* root

**DOI:** 10.1186/0717-6287-47-36

**Published:** 2014-08-19

**Authors:** Md Golam Kabir, Md Monsor Rahman, Nazim Uddin Ahmed, Md Fakruddin, Saiful Islam, Reaz Mohammad Mazumdar

**Affiliations:** Department of Biochemistry and Molecular biology, University of Chittagong, chittagong, 4331 Bangladesh; Bangladesh Council of Scientific and Industrial Research (BCSIR) Laboratories Chittagong, Chittagong, 4220 Bangladesh; Institute of Food Science & Technology (IFST), Bangladesh Council of Scientific and Industrial Research (BCSIR), Dhaka, 1205 Bangladesh

**Keywords:** *Solena amplexicaulis*, Antioxidant, Antimicrobial, Acute toxicity

## Abstract

**Background:**

This study was subjected to investigate different pharmacological properties of ethanol extract of *Solena amplexicaulis* root.

**Results:**

The extract contains flavonoid, alkaloid, saponin and steroid compounds. The extract exhibited excellent antioxidant activity in DPPH radical scavenging activity. The extract also showed potent activity in brine shrimp lethality bioassay. The LC_50_ value was found to 44.677 μg/ml. The extract showed better anti-bacterial activity against gram-negative bacteria. In antifungal assay, the maximum 79.31% of anti-mycotic activity was observed against *Aspergillus ochraceus* while minimum 44.2% against *Rhizopus oryzae.* MIC value ranged between 1500–3000 μg/ml. The extract was found moderately toxic with a 24-hr LD_50_ value of 81.47 mg/kg in Swiss albino mice. The degree of inhibition by the ethanolic extract of the root was found less than that of standard analgesic drug diclofenac sodium. The extract also showed moderate anti-inflammatory and antinociceptive activity and anti-diabetic property. Reducing power of the extract was comparable with standard ascorbic acid. Moderate in vitro thrombolytic activity, lipid peroxidation inhibition property, metal chelating ability and stress-protective activity was also observed.

**Conclusion:**

Ethanol extract of *Solena amplexicaulis* root can be valuable for treatment of different diseases.

## Background

Plants are capable of synthesizing secondary metabolites [1] which can be used for the new drugs discovery and development. Medicinal plant extracts are potential source for the development of new agents effective against infections currently difficult to treat. Antimicrobial activity of different herbal extracts has been reported against many human pathogens [2–4]. Recently, 546 species have been identified as having medicinal properties and therapeutic use [5]. The medicinal values of those plants possess some chemical active substances that produce a definite physiological action on the human body and animal health. The most important bioactive substances are alkaloid, tannin, flavonoid and phenolic compounds [6]. Drugs which are used presently for the management of pain and inflammatory conditions are either steroidal like corticosteroids or non steroidal like aspirin. All of these drugs possess more or less side and toxic effects like renal failure, allergic reactions, hearing loss or they may increase the risk of hemorrhage by affecting platelet function [7]. *Solena amplexicaulis* is a perennial dioecious climber with tuberous root found throughout Asia mainly growing in hilly dry deciduous forests, scrub jungles. The tubers, leaves and seeds are extensively used in traditional system for various ailments like hepatosplenomegaly, spermatorrhoea, appetizer, cardiotonic, diuretic and thermogenic, haemorrhoids and invigorating [8–10]. The leaves have good anti-inflammatory activity and also prescribed for skin lesions and other skin diseases [11]. The whole plant is determined to be a potential source of natural antioxidant activity [12, 13] and also used for the treatment of diabetes [14]. It is distributed in China, India, Malaysia, Myanmar, Nepal, Pakistan, and Srilanka. In Bangladesh this species is found in Chittagong, Chittagong Hill Tracts, [15]. Except anti-inflammatory activity [16] antioxidant [12] and antibacterial [17] of *Solena amplexicaulis* there is no published work on the pharmacological effect of root extracts of the plant. The previous studies were done with leaf and stem or the whole plant*.* Hence, the present study was considered worthwhile to determine the antioxidant, antimicrobial, cytotoxic, toxic and analgesic properties of crude extract of the root of the plant.

## Results

### Phytochemical screening

Qualitative phytochemical tests of *Solena amplexicaulis* were performed for the ethanol extract of the root. The results of various chemical tests for the detection and identification of chemical constituents were summarized in Table 1.

**Table 1:** **Result of phytochemical screening of ethanol root extract of** ***S. amplexiculis***

Tests	SAET	Tests	SAET
Flavonoid	+	Carbohydrate	+
Tannin	-	Resin	+
Glycoside	+	Protein	+
Alkaloid	+	Saponins	+
Anthraruinone	-	Steroids	+

(SAET denote for ethanolic extracts of *Solena amplexicaulis* root*;* (+) = present; (−) = Absent).

### Total phenol and flavonoid content and total ascorbic acid content

The total phenol and total flavonoid contents of *Solena amplexicaulis* root of ethanol extract were expressed in Gallic acid and Quercetin equivalents respectively. Total phenolic content was 12.36 mg/g Gallic acid equivalent and total flavonoid content was 18.9 mg/g Quercetin equivalent. Total ascorbic acid content was found to be 0.357 mg AA/g.

### Antioxidant activity

**Figure 1:**
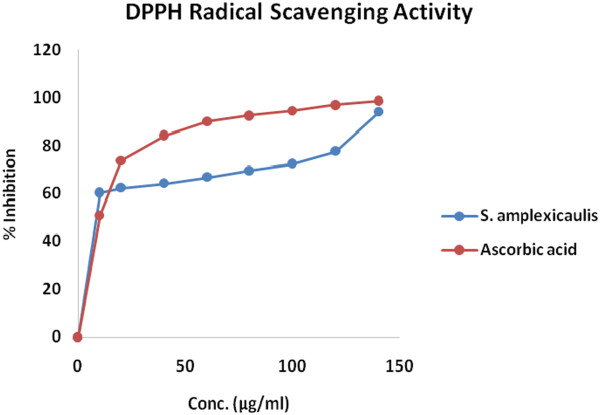
**DPPH radical scavenging activity of** ***Solena amplexiculis*** **root extract.**

The DPPH free radical scavenging activity of *Solena amplexicaulis* root ethanol extracts, and ascorbic acid is shown in Table 1. Among the eight different concentration used in the study (10, 50, 100, 200, 400, 600, 800 and 1000 μg/ml) ascorbic acid showed 50.78%, 73.62%, 84.27%, 90.13%, 92.52%, 94.52%, 97.06% and 98.66% scavenging activity where highest scavenging activity was 98.66% at concentration 1000 μg/ml. On the other hand, *Solena amplexicaulis* root ethanol extract showed 60.29%, 62.21%, 64.14%, 66.79%, 69.46%, 72.37%, 77.60%, 93.97% scavenging activity at the aforesaid eight different concentrations where highest scavenging activity of *Solena amplexicaulis* root ethanol extracts were 93.97% (1000 μg/ml). Scavenging activity (%) or % of inhibition was plotted against log concentration and from the graph IC_50_ (Inhibition concentration 50) value was calculated by linear regression analysis. IC_50_ value of ascorbic acid and *Solena amplexicaulis* root ethanol extracts were 5.46 and 4.18 respectively (Figure 1).

### Antibacterial activity

The results of antibacterial activity of the ethanolic root-extract of the plant *Solena amplexicaulis* are presented in Table 2. In comparison to reference standard tetracycline (30 μg/disc), the ethanolic root-extract exhibited moderate antibacterial activity against selected organisms.

**Table 2:** ***In vitro*** **anti-bacterial effect of ethanolic root-extract of** ***Solena amplexicaulis***

Test organism	Source ID	Diameter of zone of inhibition (mm)		
	(ATCC)	Ethanolic extract of the root from		Tetracycline 30 μg/disc
		2 mg/disc	4 mg/disc	
Gram positive				
*Bacillus subtilis*	11774	7	10	21
*Staphylococcus aureus*	25923	7.5	10	24
*Bacillus cereus*	10876	7	10.5	12
*B. polymyxa.*	842	8.5	11.0	21
*B .megaterium*	13578	7	11.5	28
*Enterococcus faecalis*	29212	6.5	9.5	19
Gram negative				
*Klebsiella pneumoniae*	65154	11.5	16.5	22
*Salmonella typhi*	12022	12.0	18.5	27
*Shigella flexneri*	13883	10.5	14.5	15
*Shigella sonnei*	8992	10	14.5	14
*Proteus vulgaris*	13315	11	15.5	17
*E. coli*	25922	13.5	18	19
*Vibrio cholerae*	15748	12.5	22.5	26
*Pseudomonas aeruginosa*	25853	12	19.5	21

### Antifungal activity

The antifungal effect of the ethanolic root extract of *Solena amplexicaulis* in various concentrations is presented in Table 3. The strongest antifungal effect was shown by the plant root extract with regard to *Aspergillus ochraceus* and *A. niger,* as the percent of inhibition was 79.31 & 62.10 respectively at 5 mg/plate. More weakly expressed was its effect with regard to *A. ustus.*

**Table 3:** **Antifungal effect of ethanolic extract of the root from** ***Solena amplexicaulis***

Organism	Source	ID	% inhibition		
			SAET (3 mg)	SAET (5 mg)	Fluconazole (100 μg)
*Aspergillus ustus*	DSM	63535	16	52	45
*Aspergillus niger*	DSM	737	47.37	62.10	65
*Aspergillus ochraceus*	DSM	824	34.48	79.31	41
*Penicillium chrysogenum*	DSM	1075	23.58	45.6	48
*Rhizopus oryzae*	DSM	2200	19.7	44.2	46

### MIC of the extract

The MIC value against Gram positive and Gram-negative bacteria ranged from 2,000 to 3,500 μg/ml, respectively (Table 4). Lowest MIC value (1500 μg/ml) of SAET was against *Bacillus polymyxa, Salmonella typhi, Shigella sonnei* and *E. coli*. MIC value was higher (3000 μg/ml) against *Bacillus cereus, Aspergillus niger and Penicillium chrysogenum*. MIC value was much higher than control antibiotic, Doxycycline hydrochloride. Considering the impurities and complex composition of the extract, MIC value against the pathogens included in this study was promising.

**Table 4:** **Minimum Inhibitory Concentration (MIC) of SAET**

Test organism	Source ID (ATCC)	MIC value (μg/ml)	
		SAET	Doxycycline hydrochloride
Gram positive			
*Bacillus subtilis*	11774	2500	5.0
*Staphylococcus aureus*	25923	2000	5.5
*Bacillus cereus*	10876	3000	4.5
*B. polymyxa.*	842	1500	6.0
*B .megaterium*	13578	2000	5.5
*Enterococcus faecalis*	29212	2500	4.5
Gram Negative			
*Klebsiella pneumoniae*	65154	2000	5.5
*Salmonella typhi*	12022	1500	6.0
*Shigella flexneri*	13883	2000	6.5
*Shigella sonnei*	8992	1500	5.5
*Proteus vulgaris*	13315	2500	4.5
*E. coli*	25922	1500	5.0
*Vibrio cholerae*	15748	2500	5.0
*Pseudomonas aeruginosa*	25853	2000	4.5
Fungi			Fluconazole (100 μg)
*Aspergill usustus*	DSM 63535	2500	7.5
*A. niger*	DSM 737	3000	7.0
*A. ochraceus*	DSM 824	2500	8.0
*Penicillium chrysogenum*	DSM 1075	3000	6.5
*Rhizopus oryzae*	DSM 2200	2500	6.0

### Assay for cytotoxicity

**Figure 2:**
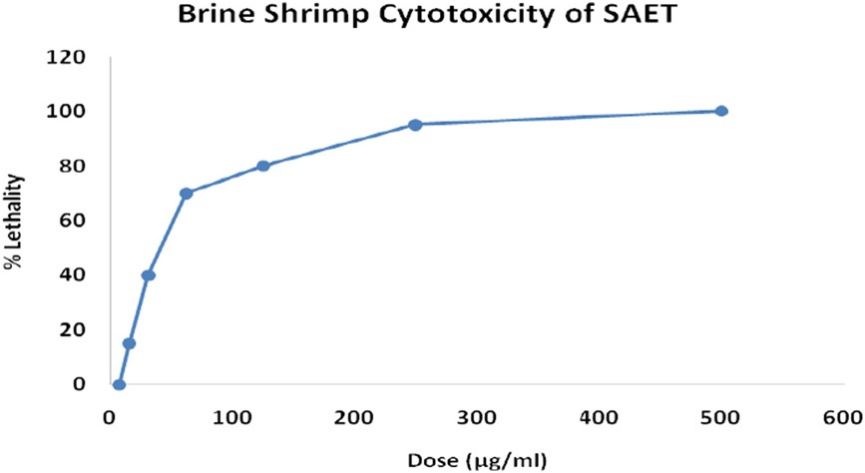
**Cytotoxicity of** ***S. amplesiculis*** **root ethanol extract (SAET).**

For the determination of cytotoxicity by Brine shrimp lethality bioassay, seven different concentrations (7.81, 15.63, 31.25, 62.5, 125, 250, and 500 μg/ml) of the ethanolic extract of *Solena amplexicaulis* root were used. The ethanolic root extract showed lethality in a dose dependent manner (Figure 2). “BioStat −2007” computer software was used for the calculation of probits for each concentration. Probits obtained by means of Finney method were then plotted against corresponding root-extract log concentration and from the plot LC_50_ (log concentration 50) value was calculated by regression analysis. LC_50_ value of the root-extract of *Solena amplexicaulis* was found 44.677 μg/ml with 95% confidence limit with lower limit 32.354 and upper limit 57.927 μg/ml.

### Acute toxicity

**Figure 3:**
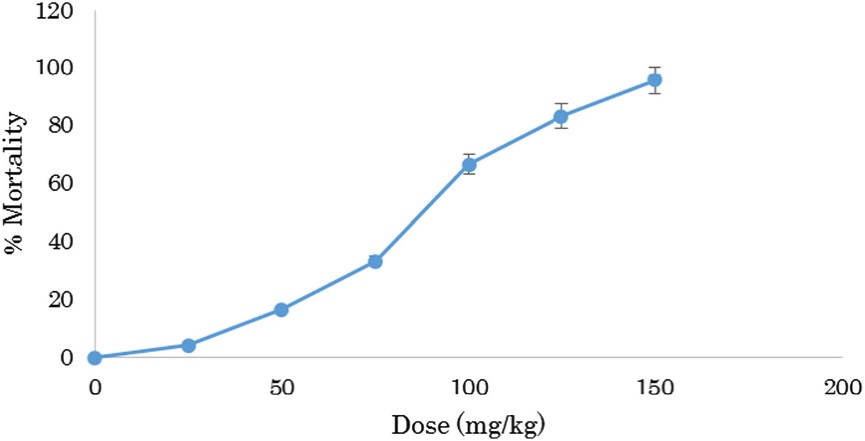
**Acute toxicity of SAET.**

Based on the mortality data 24 hours after the treatment, the LD_50_ value of the test substance for mice was found 81.54 mg/kg bodyweight with the confidence limit of 59.13-101.98 mg/kg bodyweight at 5% level of significance. The regression line equation was found as Y = − 7.1289 + 6.3633*Log_10_ (Dose) where Y was the probit mortality of the experimental mice (Table 5). Figure 3 shows acute toxicity pattern of SAET with regard to increasing concentration.

**Table 5:** **Calculation of LD**_**50**_ **value, regression equation and confidence limit by probit analysis**

log _10_ LD _50_	LD _50_ (mg/kg)	95% confidence limit (mg/kg)	Regression equation	Chi-square value	
				Calculated	Tabulated
1.91	81.54	59.13-101.98	Y = − 7.129 + 6.363*X	8.24	9.49

Here, Y and X indicate probit and Log_10_ (Dose) respectively , * mean multiplication.

### Analgesic action

**Figure 4:**
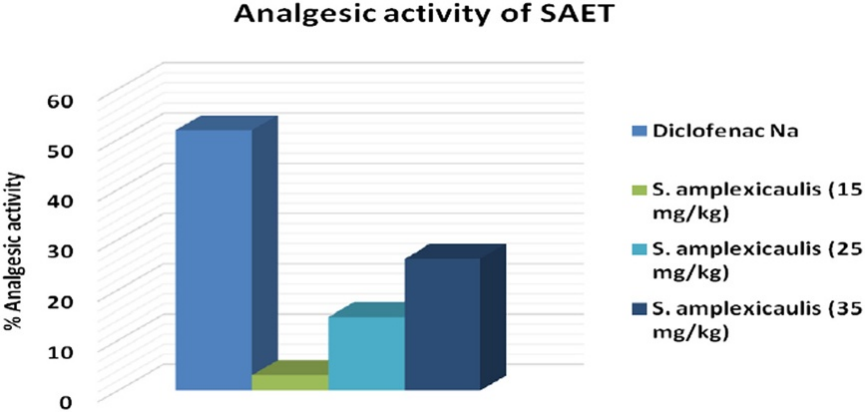
**Effect of ethanolic root-extract of** ***Solena amplexicaulis*** **on acetic acid induced writhing response mice model.**

Acetic acid induced writhing response model were used for the assay of analgesic activity of *Solena amplexicaulis* roots ethanol extract. After injecting of 1% ( v/v) acetic acid solution (3.3 ml/kg body weight) intra-peritoneally in mice, the control animal showed 82 writhing count/20 minutes but, administration of diclofenac sodium resulted in significant reduction of writhing count, from 82 to 39.5. On the other hand, ethanolic extract of the root from *Solena amplexicaulis* abridged the writhing count, from 82 to 60.5, 70, and 79.5 at the dose of 35 mg/kg bodyweight of mice, 25 mg/kg bodyweight of mice, and 15 mg/kg bodyweight of mice respectively (Table 6). The effect of the root extract and diclofenac sodium was statistically analyzed by Student’s t test. The treatment of animal with the root extract (35 mg/kg, 25 mg/kg, and 15 mg/kg) and diclofenac sodium was found significant (P < 0.001) as compared to control group. The degree of inhibition of *Solena amplexicaulis* roots extract (26.22%, 14.63%, and 3.05%) was found less than that of diclofenac sodium (51.83%) (Figure 4).

**Table 6:** **acetic acid induced writhing response of the plant root extract in mice**

Writhing count/20 minutes	Control	Diclofenac sodium	***Solena amplexicaulis***		
		(40 mg/kg)	35 mg/kg bodyweight	25 mg/kg bodyweight	15 mg/kg bodyweight
	82 ± 1.52	39.5 ± 1.34**	60.5 ± 1.48**	70 ± 1.21**	79.5 ± 1.02**

**p < 0.001, Here, All the value are expressed as Mean ± SEM.

### Anti-inflammatory activity

*Solena amplexicaulis* produced a potent anti-inflammatory activity against the paw edema in Swiss albino rats when compared with reference standards the potency was found to be inversely proportional to the time (Table 7) taken for reduction in the paw volume.

**Table 7:** **Anti-inflammatory activity of SAET**

Groups	Dose of extract (mg/kg) p.o.	Change in paw thickness (mm) ± SD (% inhibition)			
		1 hour	2 hour	3 hour	4 hour
Carrageenan control		1.34 ± 0.1	2.3 ± 0.118	3.64 ± 0.147	3.23 ± 0.161
(.01 ml of 1% w/v)					
Carrageenan	200	1.14 ± 0.103 (14.93%)	1.74 ± 0.217 (26.44%)a	2.50 ± 0.106 (31.3%)a	1.97 ± 0.115 (39.0%)a
(0.1 ml of 1% w/v) + aqueous extract					
Carrageenan	400	1.00 ± 0.12 (25.6%)	1.54 ± 0.163 (35.02%)a	1.81 ± 0.009 (50.09%)a	1.41 ± 0.188 (56.34%)a
(0.1 ml of 1% w/v) + aqueous extract					
Carrageenan	10	0.59 ± 0.118 (55.97%)a	0.8 ± 0.121 (66.24%)a	1.14 ± 0.121 (68.82%)a	0.9 ± 0.118 (72.13%)a
(0.1 ml of 1% w/v) + diclofenac sodium					

All values are expressed as mean ± SD; P < 0.05.

### Antinociceptive activity

**Figure 5:**
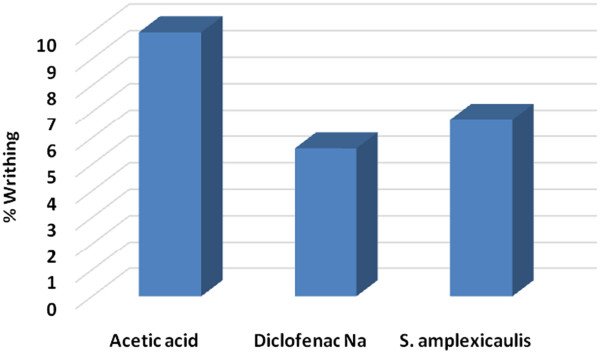
**Antinociceptive activity of SAET.**

The extract produced significant writhing inhibition comparable to the standard drug diclofenac sodium (Figure 5). The results of the test showed that SAET 500 mg/kg exhibit highly significant (P < 0.001) inhibition of writhing reflex by 6.7% while the standard drug diclofenac inhibition was found to be 5.6% at a dose of 25 mg/kg body weight. Results suggest that ethanolic root extract of *S. amplexicaulis* might possess antinociceptive activity.

### α-Amylase inhibitory property

In an array to explore the anti-diabetic activity, ethanolic root extract of *Solena amplexicaulis* was screened for the *α*-amylase inhibitory property. Ethanol extracthas significantly higher percentage of *α*-amylase inhibition, that is, 41.3% and 62.58% at 25 and 50 *μ*g/mL, respectively, while for acarbose of same concentration, the value was 33.25% and 49.57%.

### Reducing power

**Figure 6:**
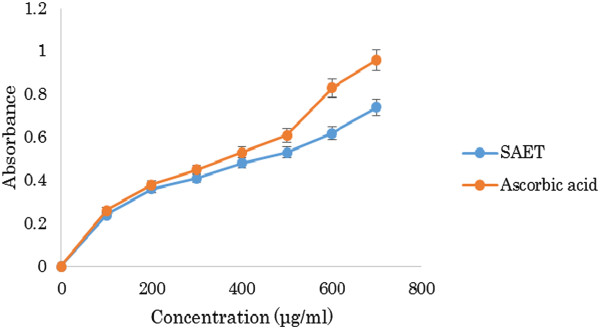
**Reducing power of SAET.**

The reducing power of the extracts was moderately strong while increasing dose it shows little increment. At 100 μg/ml concentration, reducing power of SAET was 0.24 whereas that of ascorbic acid was 0.26. But, with increasing concentration (200–400 μg/ml), reducing power of SAET increased from 0.36 to 0.48 whereas that of ascorbic acid increased from 0.38 to 0.53 (Figure 6).

### In vitro thrombolytic activity

**Figure 7:**
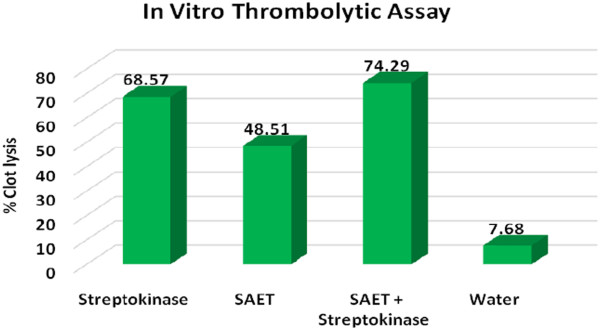
***In vitro*** **Clot lysis by Streptokinae,** ***Solena amplexicaulis*** **,** ***Solena amplexicaulis*** **in combination with Streptokinase and water.**

Streptokinase (SK) (positive control) showed 68.57 ± 1.67% clot lysis while sterile distilled water (negative control) showed only negligible clot lysis (7.68 ± 0.578%). The mean difference in clot lysis percentage between positive and negative control was very significant (p value < 0.0001). Ethanolic root extract of *S. amplexicaulis* showed 48.51 ± 1.25% clot lysis and *S. amplexiculis* in combination with Streptokinase showed 74.29 ± 0.79% clot lysis activity. Percent clot lysis obtained after treating clots with herb and appropriate controls is shown in Figure 7.

### Lipid peroxidation inhibition assay

**Figure 8:**
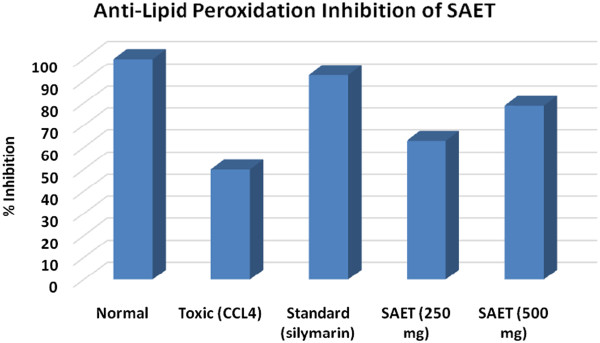
**Lipid peroxidation inhibition of SAET.**

The lipid peroxides scavenging activity of SAET was investigated and compared with standard (Salymarin). At a concentration of 250 μg/ml, the % inhibition was 62.89% and at 500 μg/ml, the %inhibition was 783.84% while inhibition of CCl_4_ and Salymarin was 49.95% and 92.77% respectively (Figure 8).

### Metal chelating effect

The chelating effect on the ferrous ions by ethanolic root extract of *S. amplexicaulis* is presented in Table 8. The extracts exhibited the ability to chelate metal ions. Metal chelating activity of SAET at 1000 μg/ml concentration was 29.35 ± 0.65 compared to 53.62 ± 0.25 of standard EDTA of Same Concentration. At 5000 μg/ml concentration, % inhibition of SAET was 41.65 ± 0.44 whereas that of standard EDTA was 94.67 ± 0.18.

**Table 8:** **Ferrous iron chelating assay of ethanolic root extract of** ***Solena amplexicaulis*** **and the standard, EDTA**

SL	Sample concentration (μg/ml)	% inhibition	
		Standard EDTA	SAET
1	1000	53.62 ± 0.25	29.35 ± 0.65*
2	2000	68.45 ± 0.35	32.71 ± 0.46*
3	3000	79.85 ± 0.13	35.15 ± 0.39*
4	4000	89.48 ± 0.23	38.23 ± 0.31*
5	5000	94.67 ± 0.18	41.65 ± 0.44*

Values are expressed as mean ± SD, *significantly different (P < 0.05).

### Spot assay

The growth of yeast cells with prior exposure to H_2_O_2_ (2 mM) in presence and absence of SAET showed significant growth difference and extract treated cells grow normally in presence of stress compounds compared to growth of control cells. This shows that the protective effect of SAET may be attributed to the presence of various anti-oxidant compounds Table 9.

**Table 9:** **Protective activity of SAET against oxidative stress**

Treatment	% survival ^a^
Untreated cells (control)	100 ± 1.52
4 mM H_2_0_2_	26.80 ± 0.58 ^b^
H_2_0_2_ + 200 μg/mL extract	39.17 ± 2.08 ^c^
H_2_0_2_ + 400 μg/mL extract	65.98 ± 1.15 ^c^
H_2_0_2_ + 800 μg/mL extract	88.66 ± 0.58 ^c^

^**a**^Average ± SD. ^**b**^Significantly different from the untreated cells (control) by the analysis of variance (ANOVA) and Dunnett’s t-test (p ≤ 0.05). ^**c**^Significantly different from the stressing agents by the analysis of variance (ANOVA) and Dunnett’s t-test (p ≤ 0.05).

## Discussion

Plants produce a lot of secondary compounds as natural protection against microbial and insect attack. Some of these compounds are toxic to animals, but others may not be toxic. Indeed, many of these compounds have been used in the form of whole plants or plant extracts for food or medical applications in human [18] because plants are the natural reservoir of many antimicrobial, anticancer agents, analgesics, anti-diarrheal, antifungal as well as various therapeutic activities [19]. The total phenol of *Solena amplexiculis* of ethanol extract were 12.36 mg/g Gallic acid equivalent and total flavonoid content was 18.9 mg/g Quercetin equivalent. Total ascorbic acid content was found to be 0.357 mg AA/g. Acceptance of medicines from such plant origin as an alternative form of healthcare is increasing because they are serving as promising sources of novel antibiotic prototypes [20]. Some of the phytochemical compounds e.g. glycoside, saponin, tannin, flavonoids, terpenoid, alkaloids, have variously been reported to have antimicrobial activity [18].

Free radicals from oxidative stress are involved in many disorders like atherosclerosis, angina pectoris, neurodegenerative diseases and cancer. Antioxidants due to their scavenging activity are useful for the management of those diseases. DPPH assay is one of the most widely used methods for screening antioxidant activity of plant extracts. The quantitative determination of antioxidants explored that high quantity of scavenging substances are found to be [21] in *Solena amplexicaulis* which plays the key role in showing free radical scavenging activity of this plant. *Solena amplexicaulis* root exhibited significant DPPH free radical scavenging activity with IC_50_ value of 4.18 μg/ml. Reducing power of *Solena amplexicaulis* is also moderately strong. Venkateshwarlu et al. [12] reported the presence of phenolic (gallic acid content is 63.63 μg/mg, respectively), and flavonoid compounds (41.18 μg/mg) present in *Solena amplexicaulis*. The phenolic compounds may contribute directly to the antioxidative action [22]. The results obtained in the present study indicate that the SAET is a potential source of natural antioxidant activity which is in accordance with the study of Venkateshwarlu et al. [12].

Crude extract at the concentration of 1 mg/disc showed 7, 7.5 and 7 mm zone of inhibition diameter against Gram-positive *Bacillus subtilis, Bacillus cereus,* and *Bacillus megaterium,* respectively and 7 mm diameter against Gram-negative *Shigella sonnei*. On the other hand, standard antibiotic Tetracycline (30 μg/disc) showed significant antibacterial activity against all tested gram positive and Gram-negative bacteria except *proteus vulgaris*. Results implicated that the Gram-positive bacteria were more sensitive to the extract than the gram-negative bacteria. *Bacillus* spp. were the most susceptible bacteria in this study. In a previous study, ethanolic stem bark extract of *Clausena heptaphylla* showed 5.5, 5.9 and 6.4 mm zone of inhibition diameter at the concentration of 2 mg/disc against Gram-positive *Bacillus subtilis, Bacillus cereus* and *Bacillus megaterium,* respectively and 3.1, 2.5, 1.8, 3.6 mm diameter against Gram-negative *Salmonella typhi, Shigella flexneri, Proteus vulgaris* and *E. coli.*[23]. Stem bark extract showed significant zone of inhibition against *Aspergillus ustus, Aspergillus niger* and *Aspergillus ochraceus* in antifungal assay. *Solena amplexicaulis* showed zone of inhibition to almost all the strains (at dose 3 and 5 mg/disc). *Solena amplexicaulis* has almost similar activity as *Clausena heptaphylla* in this study [24]. The present study justifies the claimed uses of *Solena amplexicaulis* in the traditional system of medicine to treat various infectious diseases caused by the microbes. *Solena amplexicaulis* showed a significant degree of anti-fungal activity (Table 3). The maximum anti-mycotic activity 79.31% was shown against *A. ochraceus.* Plant natural compounds are important source of mycotoxic compounds and they may provide a renewable source of useful fungicides that can be utilized in anti-mycotic drugs against infection of *A. ochraceus.* The effect of extract against *A. niger* was also higher implying that this plant can be utilized as anti-mycotic drugs against infection of *A. niger* in patients with pulmonary tuberculosis [24]. Moderate anti-mycotic effect was found against *Aspergillus ustus* at the concentration of 3 and 5 mg/ml. Fluconazole was used as standard antifungal agent to compare the potentials of extract. There are, however, alarming reports of opportunistic fungal infections which describe that the resistance of the organisms increased due to indiscriminate use of commercial anti-microbial drugs commonly used for the treatment of infectious disease. This situation forced the researchers to search for new anti-microbial substance from various sources including medicinal plant [24]. Karthika and Paulsamy [17] reported that *S. Amplexicaulis* extracts of leaf and stem exhibited antibacterial and anti-fungal activity. The findings revealed that medicinal plant *Solena amplexicaulis* can play a vital role in combating fungal resistance.

Lowest MIC value (1500 μg/ml) against *B. polymyxa, Salmonella typhi, Shigella sonnei* and *E. coli* was showed by the extract. Lowest MIC value (2500 μg/ml) was found against fungi such as *Aspergillus ustus*, *A. ochraceus and Rhizopus oryzae*. MIC value of the extract against the test pathogens are much higher than that of Doxycycline hydrochloride which may be due to the crude nature of the pigment extracts. Natural compounds from plants are important source of mycotoxic compounds and they can be a source of useful fungicides that can be utilized in anti-mycotic drugs against infection of *A. ochraceus*[24].

Different pharmacological properties can be assumed on the basis of brine shrimp cytotoxicity of any plant extract [23]. In this research, six different concentrations (7.81, 15.62, 31.25, 62.5,125, 250, 500 μg/ml) of *Solena amplexicaulis* extract were used to determine its cytotoxicity by brine shrimp lethality bioassay. The extract showed lethality in a dose dependent manner. LC_50_ value of *Solena amplexicaulis* ethanol extract was found 44.68 μg/ml (Figure 2). The LC_50_ value found in this study was very significant suggesting the ethanol extract of *Solena amplexicaulis* has high potentiality to kill cancer cells as well as pests [25]. This significant lethality of the crude plant extract (as LC_50_ value less than 100 μg/ml) to brine shrimp is indicative of the presence of potent cytotoxic compounds which warrants further investigation.

From the study, it was observed that the water-soluble absolute ethanolic extract of the root of *Solena amplexicaulis* (Gandhi) exhibited toxic properties to the experimental animals (Swiss albino mice). The extract showed its acute toxicity on mice when administered orally in the range of 50 to 150 mg/kg bodyweight of mice. The 24-hr LD_50_ values were found to be 81.54 mg/kg bodyweight of mice. However, it caused no mortality in the treated mice and rats when they were treated orally at the doses of 25 mg/kg and 75 mg/kg bodyweight respectably, though some physiological changes occurred. It might have the properties of muscarinic activity and respiratory analeptics on mice and rats. It might also cause visceral changes in mice as well as possess central depressant property to a variable extent on mice. Thus, the study presented here indicates the antinociceptive action of the ethanolic root extract in the acetic acid writhing test could be due to the inhibition of the release of TNF-α, interleukin-1β and interleukin-8 [26] by resident peritoneal cells. The root extract of the plant showed significant inhibition (P < 0.001) of acetic acid induced writhing response of mice. So it can be suggested that *Solena amplexicaulis root* extract has potential analgesic action [27].

In the present study, pre-treated oral administration of extract was effective in reducing the oedematogenic response evoked by carrageenan in rats between the 2nd and 4th hours after the injection. This evidence allows us to suggest that anti-inflammatory actions of the extract are related to the inhibition of one or more intracellular signaling pathways involved in the effects of several inflammatory mediators. Anti-inflammatory activity increased with the increase in the dose of the extract. Significant anti-inflammatory activity of SAET also corresponds to the presence of flavonoid compounds in the extract as flavonoids are considered to possess anti-inflammatory activity [28].

SAET showed significant antinociceptive activity compared to diclofenac sodium. Acetic acid-induced writhing model represents pain sensation by triggering localized inflammatory response. Acetic acid, which is used to induce writhing, causes algesia by liberation of endogenous substances, which in turn excitethe pain nerve endings [29] localized inflammatory response.

Leaf and stem extracts of *S. amplexicaulis* has been reported to possess anti-diabetic activity in terms of their *α*-amylase inhibition activity [30, 31]. Ethanolic root extract also showed better anti-*α*-amylase property (62.58% at 50 *μ*g/mL) than standard drug acarbose (49.57% at 50 *μ*g/mL). This enzyme inhibitory activity of SAET may be attributed to presence of different active compounds such as flavonoids, glycosides and polyphenols.

In the present study, the reducing power of the SAET was evaluated measuring the conversion of a Fe^3+^ ferricyanide complex to the ferrous form. 

The reducing power of the investigated extract linearly increased with concentration. At 100 μg/ml concentration, reducing power of SAET was 0.24 whereas that of ascorbic acid was 0.26. In vitro thrombolytic activity of SAET is significant with 48.51% clot lysis capability compared to that of Streptokinase (68.57%). It can be considered for compound isolation in order to detect future anti-tumour compounds.

*In vitro* thrombolytic activity of SAET is significant with 48.51% clot lysis capability compared to that of Streptokinase (68.57%). It can be considered for compound isolation in order to design future anti-tumour drug. SAET had appreciable lipid peroxidation inhibition activity (Figure 8). At a concentration of 250 μg/ml, the% inhibition of Lipid peroxidationwas 62.89% and at 500 μg/ml, the % inhibition was 78.84% while % inhibition of CCl_4_ and Salymarin was 49.95% and 92.77% respectively. Elevated lipid peroxidation is a common trait of cancer [32] and results suggest SAET can be used in cancer for its anti-lipid peroxidation capability and can protect cells chain reaction from breaking down due to lipid peroxidation.

Non-ligand or poorly liganded iron may be the basis of many diseases involving cell death and apoptosis [33]. Thus, the chelating ability of the extracts in regards to ferrous ions was investigated. Even though the extracts were not as strong Fe^2+^ chelators as EDTA, the investigated extract demonstrated notable chelating properties. Ferrous ion chelating activity of SAET was close to the results of Karthika *et al.*[17]. Metal chelating capacity was significant as they reduced the concentration of catalyzing transition metal in lipid peroxidation. Spot assay result provides us an indication that SAET exert protective activity to cells against stress and agents causing DNA damage may be due to different compounds it possess with antioxidant activity.

## Conclusions

This study suggests that *Solena amplexicaulis* extract possesses some potentials in free-radical scavenging activity, cytotoxic and analgesic effect but it has low antimicrobial (antibacterial and antifungal) activity. The toxic nature of the alcohol extract is evident from the acute oral toxicity. Since, crude ethanol extract of *Solena amplexicaulis* showed antimicrobial, cytotoxic and analgesic effect, it can be assumed that different active secondary metabolites were present in this extract. These can be a strong scientific evidence to use this plant as a useful source of both biological and pharmacological references. However, further studies are necessary to elucidate the specific active compounds in the root extracts of *S. amplexicaulis.*

## Methods

### Ethical statement

All the experiments including animal used in this study was approved by the ethical review committee of BCSIR laboratories, Chittagong, Bangladesh.

### Media and chemicals

DPPH (1, 1-diphenyl, 2-picrylhydrazyl), TCA (trichloroacetic acid) and ferric chloride, Ascorbic acid was from SD Fine Chem. Ltd. India, ammonium molybdate from Merck, Germany. Mueller-Hinton broth and agar media (Hi media, India), final pH 7.3 ± 0.2 (at 25°C), was used for MIC determination and antibacterial. On the other hand, potato dextrose agar media (Hi media, India), final PH (at 25°C) 5.6 ± 0.2 and artificial seawater (3.8% NaCl solution) were used for the determination of antifungal and cytotoxic effect.

### Collection of plant materials

Very rare medicinal plant *Solena amplexicaulis* was collected from various regions of Khagrachari District of Chittagong Hill Tracts of Bangladesh. The plant was identified by Professor Dr. Md. Mostofa Kamal Pasha, Taxonomist, Department of Botany, University of Chittagong, Chittagong, Bangladesh.

### Extraction

The fresh roots of *Solena amplexicaulis* were washed with water immediately after collection and then chopped into small pieces, air-dried at room temperature for about 10 days and pulverized into powder form and stored in an airtight container. 2 kg root powder was macerated in 6 L pure ethanol for 7 days at room temperature. After 7 days, root’s ethanolic extract was filtered off through a cotton plug and finally with a Whatman No. 1 filter paper. Then the solvent retaining in the crude extract was evaporated under reduced pressure below 50°C through a rotary vacuum evaporator. The concentrated extracts were collected in a Petri dish and allow to air dry for complete evaporation of ethanol. The whole process was repeated two times and finally, 80 gm concentrated *Solena amplexicaulis* root extract (SAET) was obtained (yield 4% w/w) which was kept in refrigerator at 4°C.

### Phytochemical screening

Freshly prepared crude extracts of *Solena amplexicaulis* root were subjected to qualitative test for the presence of different constituents using the following reagents and chemicals, flavonoids with the use of Mg and HCl; tannins with ferric chloride and potassium dichromate solutions and saponins with ability to produce stable foam and steroids with Libermann Burchard reagent, reducing sugars with Benedict’s reagent and observed color change in respective [34].

### Determination of total phenolic content

Folin-Ciocalteu method was used to determine the total phenolic content; Folin-Ciocalteu oxidized the extract whereas sodium carbonate neutralized it. Blue color formed and the absorbance was measured at 760 nm after 60 min by using gallic acid (GA) as standard. Total Phenolic content was expressed as mg GA equivalent/ gm of extract [35].

### Determination of total flavonoid content

Method described by Meda et al. [36] was followed to determine the flavonoid content where quercetin was used as standard.

### Determination of total ascorbic acid (ASC)

Total ascorbic acid (ASC) content of the extract was determined according to the method described by Roe and Kuether [37].

### Antioxidant activity

The antioxidant activity of the extract is carried out based on the scavenging activity of the Table 1, 1 diphenyl-2 picrylhydrazyl (DPPH) according to the method described by Brand-William et al. [38].

### Brine shrimp lethality assay

Cytotoxic activity of plant extract was determined by Brine-Shrimp lethality bioassay as previously described [39].

### α-amylase inhibition assay

To determine the *in vitro α*-amylase inhibition by ethanolic root extracts of *Solena amplexicaulis,* the standard procedure [40] was adopted with slight modification.

### Reducing power

The reducing power of *Solena amplexicaulis* extract was determined according to the method of Oyaizu [41].

### In vitro thrombolytic activity

Individuals from whom the blood samples were collected were informed and upon their consent they were included in this study. Samples were taken from adult healthy individual. No children were included in this study. In vitro thrombolytic activity was performed according to Ratnasooriya et al. [42].

### Metal chelating effect

The chelation of ferrous ions by the extract was estimated as per the method of Dinis et al. [43].

### Antibacterial screening

The *in vitro* sensitivity of the bacteria to the test material was done by disc diffusion technique [44] against 6 gram-positive (*Bacillus subtilis* ATCC 11774*, Staphylococcus aureus* ATCC 25923*, Bacillus cereus* ATCC 10876*, Bacillus polymyxa* ATCC 842*, Bacillus megaterium* ATCC 13578*, Enterococcus faecalis* ATCC 29212) and 8 gram-negative bacteria (*Salmonella typhi* ATCC 12022*, Klebsiella pneumonia* ATCC 65154*, Shigella flexneri* ATCC 13883*, Shigella sonnei* ATCC 8992*, Proteus vulgaris* ATCC 13315*, E. coli* ATCC 25922*, Vibrio cholerae* ATCC 15748*, Pseudomonas aeruginosa* ATCC 25853). All the results were compared with the standard antibacterial drug tetracycline.

### Determination of Antifungal activity

The poisoned food technique [45] was used to screen for anti-fungal activity.

### Minimum inhibitory concentration (MIC)

Minimum Inhibitory Concentrations (MIC) were determined by macro dilution method [45].

### Spot assay

Wild type *Saccharomyces cerevisiae* was used to investigate the effect of extract on the growth of yeast cells, to select the doses of extract used in the adaptive treatments. *Saccharomyces cerevisiae* cells (OD_600_ of 0.6-1) without and with exposure to increased concentrations of extract (200, 400 and 800 μg/ml) and stressing agent (2 mM) were incubated in a YPD medium for 1 hr at 28°C /160 rpm. In all cases, about 3 μl of yeast saturated cultures after 1 hr incubation were serially diluted (10^−1^, 10^−2^, 10^−3^and 10^−4^), and were dropped on standard YPDA plates and Hydroxyurea (150 mM) laden YPDA plates. Plates were incubated at 28°C, and growth was recorded after 24, 48 and 72 hrs by counting colony. The lowest concentration was chosen which could improve cell growth compared to cohorts exposed to stress without being treated with plant extract.

### Experimental animals and diets

Swiss albino mice of both sexes weighing between 25 gm and 30 gm were taken from animal house of BCSIR laboratories, Chittagong. The animals were acclimatized to room temperature (28 ± 5°C) with a relative humidity of 55 ± 5% in a standard wire meshed plastic cages for 4 to 5 days prior to commencement of the experiment. During the entire period of study the animals were supplied with standard pellet diet and adlibitum water.

### Acute toxicity

Acute toxicity of ethanolic root extract of *S. amplexicaulis* in mice was determined according to Bulus *et al.*[46].

### Analgesic activity

Analgesic activity was performed according to Vittalrao et al. [47].

### Lipid peroxidation inhibition assay

The lipid peroxidation inhibition assay was determined according to the method described by Liu *et al.*[48].

### Antinociceptive activity

Antinociceptive activity of the crude extract was tested using the model of acetic acid induced writhing in mice [49].

### Anti-inflammatory activity

Carrageenan induced rat hind paw edema was used as the animal model of acute inflammation according to the method of Jude et al. [50].
